# EHMTI-0078. Headache attributed to masticatory myofascial pain: clinical features and management outcomes

**DOI:** 10.1186/1129-2377-15-S1-D13

**Published:** 2014-09-18

**Authors:** YM Costa, AL Porporatti, J Stuginski-Barbosa, LR Bonjardim, PCR Conti

**Affiliations:** 1 Prosthodontics, Bauru School of Dentistry University of São Paulo, Bauru, Brazil; 2 Biological Sciences, Bauru School of Dentistry University of São Paulo, Bauru, Brazil

## Introduction

To define better the association between headache and temporomandibular disorders it is important to identify if there is evidence of a headache attributed to temporomandibular disorders.

## Aims

To describe the characteristics of headache attributed to temporomandibular disorders pain and to assess the effect of two management strategies in headache intensity and frequency.

## Methods

The sample (n=60) of this randomized controlled trial was comprised of masticatory myofascial pain patients, according to the Research Diagnostic Criteria for Temporomandibular Disorders (RDC/TMD) and headache complaints and divided into two groups. The group 1 received only counseling for behavioral changes and the group 2, besides counseling, received occlusal splint. Follow-up was 5 months with three assessments moments. Outcomes were the temporomandibular disorders-related headache characteristics measured by a questionnaire, headache intensity (visual analogue scale - VAS) and frequency (questionnaire). Two-way ANOVA and Chi-Square test were used considering a 5% significance level.

## Results

The clinical features of headache attributed to temporomandibular disorders were the long duration, fronto-temporal bilateral location and tightening/pressing quality. There was a general reduction in the headache intensity and frequency without differences between groups. The baseline headache intensity mean (SD) was 7.55 (2.24) for the group 1 and 6.52 (1.63) for group 2. Final values were, respectively, 3.13 (2.19) and 2.5 (2.33).

## Conclusion

Long duration, fronto-temporal bilateral location and a tightening/pressure quality are the most distinguished characteristics in headaches secondary to masticatory myofascial pain. Besides, the management of masticatory myofascial pain could be effective in the improvement of headache attributed to temporomandibular disorders.

No conflict of interest.

